# Pseudorabies virus: a neglected zoonotic pathogen in humans?

**DOI:** 10.1080/22221751.2018.1563459

**Published:** 2019-01-21

**Authors:** Gary Wong, Jiahai Lu, Wenhong Zhang, George Fu Gao

**Affiliations:** a Institut Pasteur of Shanghai, Shanghai, People’s Republic of China; b Département de microbiologie-infectiologie et d’immunologie, Université Laval, Québec, QC, Canada; c School of Public Health, Sun Yat-Sen University, Guangzhou, Guangdong, People’s Republic of China; d Key Laboratory of Molecular Virology, Institute of Medical Microbiology, Department of Infectious Diseases, Huashan Hospital, Fudan University, Shanghai, People’s Republic of China; e CAS Key Laboratory of Pathogenic Microbiology and Immunology, Institute of Microbiology, Chinese Academy of Sciences, Beijing, People’s Republic of China; f National Institute for Viral Disease Control and Prevention, Chinese Center for Disease Control and Prevention (China CDC), Beijing, People’s Republic of China

## Introduction

Pseudorabies (also known as Aujeszky's disease) is a viral disease caused by pseudorabies virus (PRV). First isolated in 1902, classical PRV has circulated globally since the 1980s, especially in locations with large pig populations. PRV is extremely infectious and infected pigs shed large quantities of virus in bodily secretions and excretions. PRV is mainly spread via direct contact, but may also transmit by air, water and contaminated fomites. Outbreaks of PRV in pigs are difficult to control, causing catastrophic economic losses in the swine industry. Immunization of pigs with the Bartha-K61 vaccine, a live-attenuated PRV strain [1], effectively controls virus spread but does not prevent infection [2]. Targeted vaccination campaigns have eliminated classical PRV from domestic pigs (but not wild feral swine) in most of Europe, USA and New Zealand. In 2011, variant PRV strains emerged from Bartha-K61-vaccinated pig farms in China [3]. The Bartha-K61 vaccine did not effectively protect against variant PRV in pigs [4] (although this is disputed [5]), but since then, these novel viruses have continued to circulate in Northern, Eastern and Southern China.

## Phylogenetic and molecular analysis of PRV

**Figure 1.**
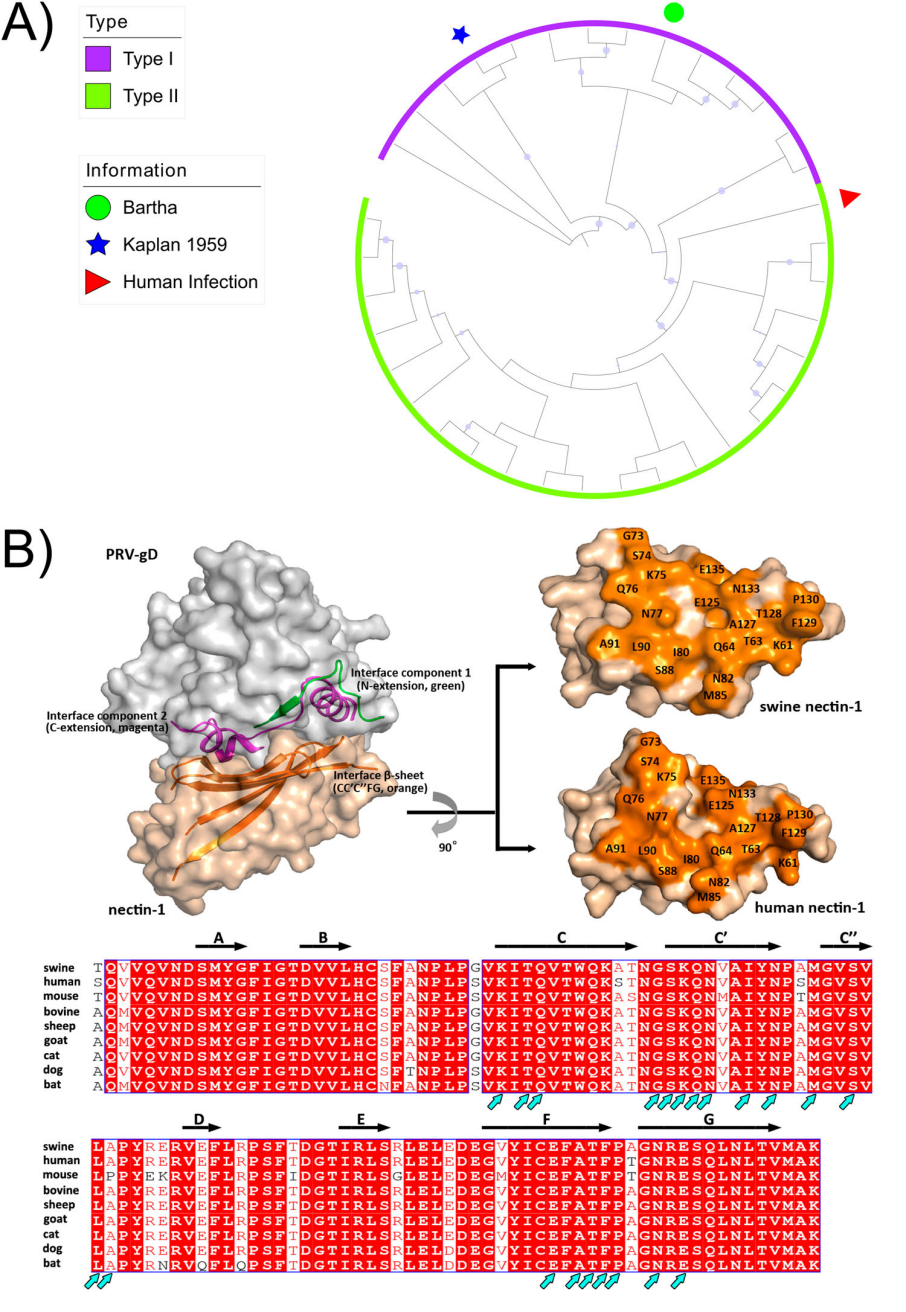
Phylogenetic and structural analysis of PRVs. (A) Phylogenetic relationship of genome sequences between PRVs isolated from human and animals. We retrieved 35 publicly available whole sequences from NCBI and partial genomes for the PRV isolated from a patient [12]. Sequences were aligned by MAFFT. After getting conserved blocks from sequence alignment by Gblocks v0.91b, RAxML v8.2.4 was used to reconstruct the phylogenetic tree under the GTR+G model, with bootstrapping of 1000 replicates. iTOL v4 was used to visualized the phylogeny. The size of the circle is proportional to the bootstrap value, and only bootstrap values ≥70 was visualized. Accession numbers of PRV sequences used in this study were as follows: BK001744.1, KU360259.1, KT983811.1, KT983810.1, MG551317.1, MG551316.1, MG589642.1, NC_006151.1, LC342744.1, KX423960.1, KU900059.1, KP722022.1, KM189913.1, KM189914.3, KT824771.1, KT809429.1, KP257591.1, KP098534.1, KM061380.1, KJ789182.1, KJ717942.1, LT934125.1, KU056477.1, KU552118.1, JQ809330.1, JQ809329.1, KU315430.1, KU057086.1, KU198433.1, KM189912.1, KC981239.1, JF797219.1, JF797218.1, JF797217.1, and JQ809328.1. (B) Structure of PRV gD binding to the nectin-1 receptor. An alignment of amino acid residues in nectin-1 of various animal species are shown, and the residues important for binding (providing 5 or more Van der Waals contacts) are denoted with arrows.

Phylogenetic analysis of publicly available genome sequences for classical and variant PRVs based on whole genome sequences (Figure 1(A)) shows that classical PRV strains from the USA and Europe, and variant PRV strains from China can be categorized into genotypes I and II, respectively. For PRV entry into host cells, viral glycoprotein gD is utilized to bind the host cell via the nectin-1 receptor, similar to herpes simplex viruses [6]. PRV gD was previously shown to engage both human and swine-origin nectin-1 with similar binding affinities, and that the following amino acid residues in the nectin-1 receptor contribute to over 90% of the total inter-molecule contacts. They are: K61, T63, Q64, K75, Q76, N77, I80, N82, M85, S88, L90, A91, E125, A127, T128, F129, P130, N133 and E135 [7]. Importantly, these residues are conserved across many different species including mice (aside from an A91P mutation), cows, sheep, goats, cats, dogs and bats (Figure 1(B)), suggesting that PRV is able to bind to and enter cells of these species, and that cross-species infection may be a possibility.

## PRV infection in animals

**Figure 2.**
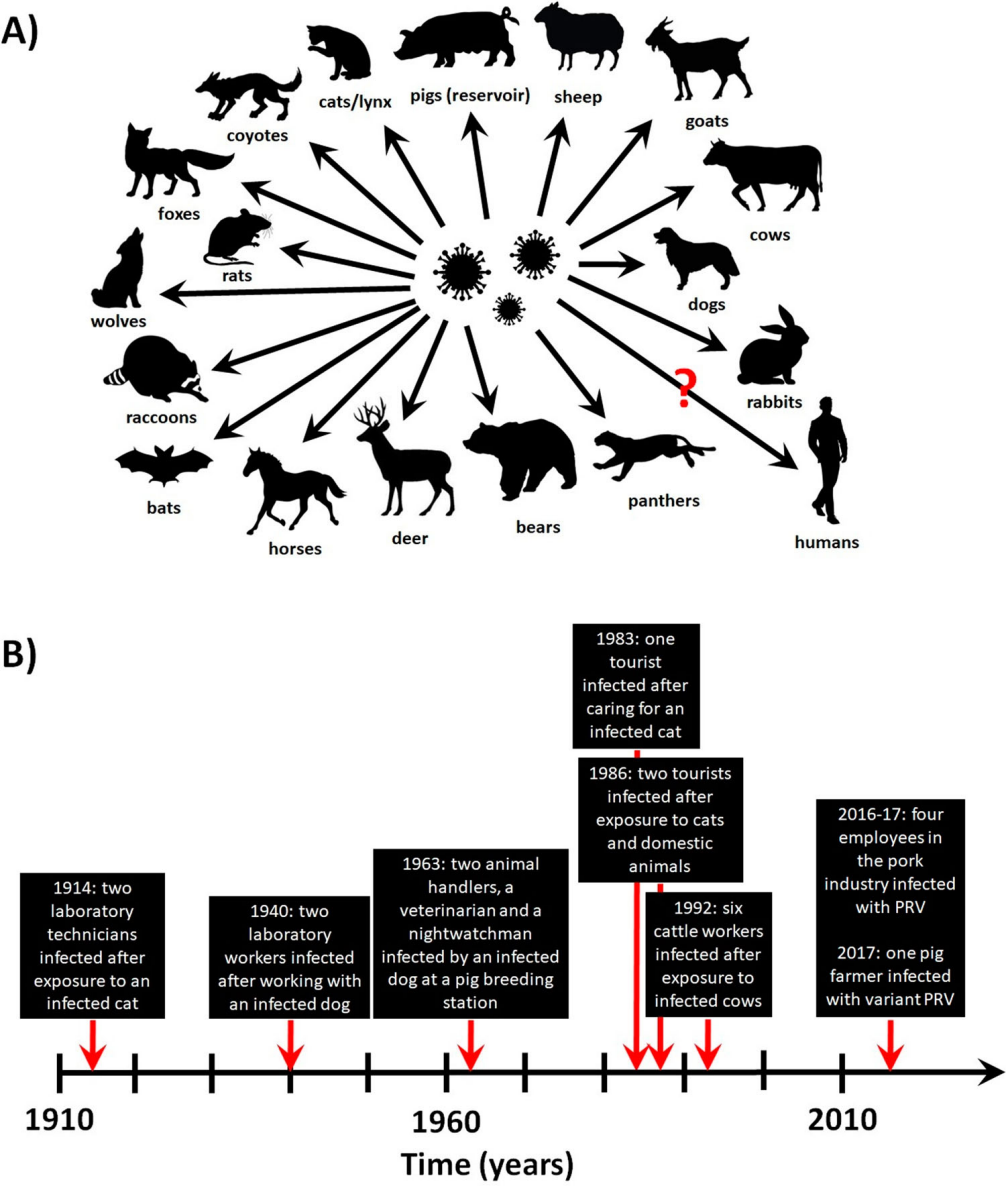
PRV infection in animals and humans. (A) Confirmed and potential (marked by a question mark) species susceptible to natural PRV infections. (B) Timeline of the reported PRV infections in humans.

Pigs are the only natural carriers of PRV, and latent infection has only been observed in these animals. After oral/nasal infection, the virus replicates in the upper respiratory tract before attacking sensory nerve endings, crossing synapses to infect neurons and invading the nervous system. The rapid pathogenesis in pigs is thought to be in part due to cell-associated viremia, in which PRV-infected monocytes and lymphocytes act as carrier cells for virus transport all over the body [8]. The clinical outcome primarily depends on the age of the animals. In younger suckling pigs, neurological signs (trembling, convulsions, incoordination, and paralysis leading to death within 24–36 h of disease onset) are common and the mortality rate is close to 100%. In older pigs, respiratory signs such as cough, sneezing, rhinitis, and laboured breathing are contrarily observed, and latent infection is established in the peripheral nervous system. Infection of pregnant swine with PRV can result in abortion or delivery of stillborn or weakened piglets that die shortly after birth. Additionally, fatal PRV infections have been reported in cats, dogs, cattle and other animals (Figure 2(A)), which typically only live for several days after disease onset [9].

## Reports of suspected PRV infection of humans

Whether humans are susceptible to PRV infection has been a subject of controversy. A past serosurvey of 455 volunteers with either suspected PRV infection or high-risk of occupational exposure were shown to be negative for PRV-specific neutralizing antibodies, and self-inoculation with 10^3.4^ TCID_50_ (intracutaneous) or 10^6.1^ TCID_50_ (subcutaneous) of PRV did not result in any symptoms [9].

Conversely, suspected human infections with classical PRV were reported as early as 1914, in which two laboratory technicians exposed to contaminated materials developed weakness, restlessness, sore throat, and itching [9]. Similar reports were noted in 1940 (two laboratory workers exposed to a diseased dog) and 1963 (four workers exposed to an infected dog at a pig breeding station) [9]. Pruritus was observed in both outbreaks but specific antibodies and live virus could not be detected or cultured from these patients. In 1986, PRV was reported in three patients in Europe [10]. All three had contact with cats and/or other domestic animals, and symptoms started approximately 1–3 weeks afterwards with fever, sweating, weakness and tiredness, before progressing to involvement with the central nervous system (including dysphagia, paraesthesia, tinnitus, etc.). Recovery was complete but slow, lasting several months to almost one year. The patients were seropositive for specific neutralizing antibodies with reciprocal titres of 8–16 when tested 5–15 months after disease onset but were seronegative at 2–24 months after testing [10]. In an unrelated incident, six out of seven workers with direct contact to cattle exhibiting PRV-induced disease also developed clinical signs, such as pruritus of the palms that spread onto the lower and upper arms, shoulders and back [11].

Recently, one case of endophthalmitis (an inflammation of the interior of the eye) was reported [12]. A 46-year-old woman from China, who worked as a pig farmer, developed a fever, headaches and visual impairment. Next-generation sequencing (NGS) of the vitreous humour sample taken from the patient showed that the most likely causative agent was variant PRV, a result supported by PCR analysis and Sanger sequencing. The phylogenetic analysis of the PRV genome sequence shows that it belonged to genotype II (Figure 1(A)). The patient was found to be seropositive for PRV but it was unclear whether this is class IgG or IgM antibodies (which would have indicated an active infection). Cerebrospinal fluid samples were negative when analysed by NGS or PCR [12]. The fever and headaches resolved within 1 month after antiviral treatment, but visual acuity did not fully recover as of 6 months after disease onset.

Another Chinese study [13] investigated four patients with severe encephalitis of unknown aetiology to identify the causative pathogen. All worked in the pork industry with exposure to raw meats. The patients presented with fever, convulsion, loss of consciousness and respiratory failure within 1–4 days after hospitalization. Analysis of cerebrospinal fluids by NGS showed two out of four patients were positive for PRV, whereas PRV-specific antibodies (unsure whether this is IgG or IgM) were present in three out of four patients. It was concluded that PRV could be a cause for severe encephalitis, and that patients with encephalitis of unknown origin should be tested for PRV to rule out infections with this pathogen [13].

## Discussion, conclusions and perspectives

Despite vaccination efforts, classical PRV is still prevalent in wild feral pigs and variant PRV in many Chinese pigs. High levels of exposure between humans and swine (especially via occupational contact) means that if PRV can infect humans, it is surprising that relatively few cases has been reported thus far. It could be speculated that due to species differences (i.e. immune responses), PRV may be less virulent, or asymptomatic, in most humans as opposed to other animals. Additionally, the likely possibility that current clinical diagnostic protocols for unexplained fevers and encephalitis do not always include the specific detection of PRV would contribute to an underreporting of cases. Advances in detection (i.e. NGS) techniques will help with identifying unknown causative pathogens from the cerebrospinal fluids of patients with meningoencephalitis [14] and may provide a clarification for at least a portion of patient cases with unexplained encephalitis or endophthalmitis. It is important to note that due to exposure of animal farmers to high levels of environmental contaminants (including many other microbes which can also cause disease), it is difficult to correlate the presence of PRV DNA sequences as detected by NGS with actual pathogenesis and clinical symptoms in the host. Thus, any preliminary NGS data needs to be supported by live virus isolation from the infected host. If this is not possible, then the presence of viral DNA or the PRV-specific IgM antibodies assays are two indicators which would support an active infection in the host.

The evidence so far suggests that PRV infection may be an occupational risk and may cause disease in humans, but live virus has not yet been isolated from patients. Live PRV has not yet been shown to cause disease in humans after infection, and obviously no re-isolation experiments are possible without live PRV from humans. Thus, not all of Koch's Postulates have been fulfilled, and despite circumstantial observations, there is still no conclusive evidence for PRV-induced disease in humans. Interestingly, past studies in experimental PRV infection of rhesus macaques showed inconsistent results [15]. A 1930 study using a strain of Aujeszky virus in England failed to establish infection via intracerebral or intramuscular inoculation, but subsequent investigations with an Iowa and a Hungarian strain were successful in producing neurological signs via intracerebral injection [15]. Additionally, pre-existing immunity to herpes B virus was found to have a protective effect against intracerebral inoculation of PRV in macaques [15]. This suggests that there may be strain-specific differences between PRV and infectivity to monkeys or humans, and that pre-existing immunity against related viruses may have an impact on the severity of disease symptoms. In future studies, virus isolation from patient samples should be attempted in both cell culture and live animals (i.e. BALB/c mice) susceptible to Aujeszky's disease. Additionally, advanced detection technologies, such as NGS, should be applied in all unexplained/suspected infections to discover novel pathogens that infect and cause disease in humans, in order to help predict future outbreaks in conjunction with serological, virological and epidemiological findings [16]. These efforts will massively contribute towards the assessment of the true public health risk potentially posed by PRV infections.
